# Effect of different bonding agents and airborne-particle abrasion on shear bond strength and mode of failure between 3D-printed denture tooth-colored specimens and 3D-printed denture bases

**DOI:** 10.1186/s12903-026-10075-1

**Published:** 2026-11-05

**Authors:** Amany Mostafa Saad Farahat, Mahmoud Amin Faty

**Affiliations:** Department of Oral and Maxillofacial Prosthodontics, Faculty of Dentistry, Ain Shams University, Organization of African Unity street, El Weili, Cairo Governorate, Cairo, Egypt

**Keywords:** Shear bond strength, Three-dimensional printing, Resin cement, Universal testing machine, Airborne-particle abrasion

## Abstract

**Background:**

In conventionally fabricated dentures, debonding between the denture teeth and the denture base accounts for 22 to 30% of all denture repairs, with failures predominantly occurring in the anterior region. Various surface treatments and bonding protocols have been proposed to enhance adhesion between denture teeth and denture base; however, reliable bonding between three-dimensional (3D)-printed denture teeth and denture bases remains a challenge, and evidence regarding the influence of bonding strategies and surface treatment protocols is limited.

**Methods:**

3D-printed tooth-colored and denture base specimens (*n* = 72) were fabricated and allocated into 3 bonding agent groups (*n* = 24); in each group, the tooth-colored specimens were subdivided into two subgroups. In subgroup A, the undersurface of the tooth-colored specimens was air-abraded, while in subgroup B, it remained untreated. The bonding agent in the first group was 3D printing denture base liquid resin; in the second group, a universal adhesive; and in the third group, a resin cement. All the specimens were subjected to thermocycling for 5000 cycles, after which shear bond strength was evaluated using a universal testing machine. The mode of failure was evaluated under a stereomicroscope. Two-way analysis of variance was used to assess the effect of the bonding agents, surface treatment, and their interactions. Pairwise comparisons were performed utilizing pairwise t-tests with Bonferroni correction. Failure mode distributions were evaluated using the Fisher’s exact test.

**Results:**

The null hypothesis was partially rejected. Two-way ANOVA revealed significant main effects of bonding agent (*P* < 0.001); neither surface treatment (*P* = 0.881) nor the bonding agent-surface treatment interaction (*P* = 0.146) reached statistical significance. The highest shear bond strength was reported in the liquid resin subgroups 9.04 ± 2.27 and 8.39 ± 2.12, and the lowest shear bond strength was in the resin cement subgroups 5.12 ± 1.75 and 4.27 ± 1.46. Airborne-particle abrasion had no significant effect within any individual bonding agent (*P* = 0.438, *P* = 0.130, *P* = 0.315, respectively). Failure mode distribution differed significantly among the bonding protocols with and without air abrasion (*P* < 0.001). Airborne-particle abrasion significantly altered failure mode distributions for liquid resin (*P* < 0.001) and universal adhesive (*P* = 0.001), but not for resin cement.

**Conclusions:**

Under the tested conditions, liquid resin showed higher mean shear bond strength than universal adhesive and resin cement for bonding 3D-printed denture tooth-colored specimens and denture base, with a predominant cohesive failure mode. The effect of airborne-particle abrasion of the tooth-colored specimen-bonding surface did not significantly change shear bond strength within any individual group, but, resulted in a statistically significant change in failure mode for both the liquid resin and universal adhesive bonding protocols.

**Graphical Abstract:**

**Figure:**
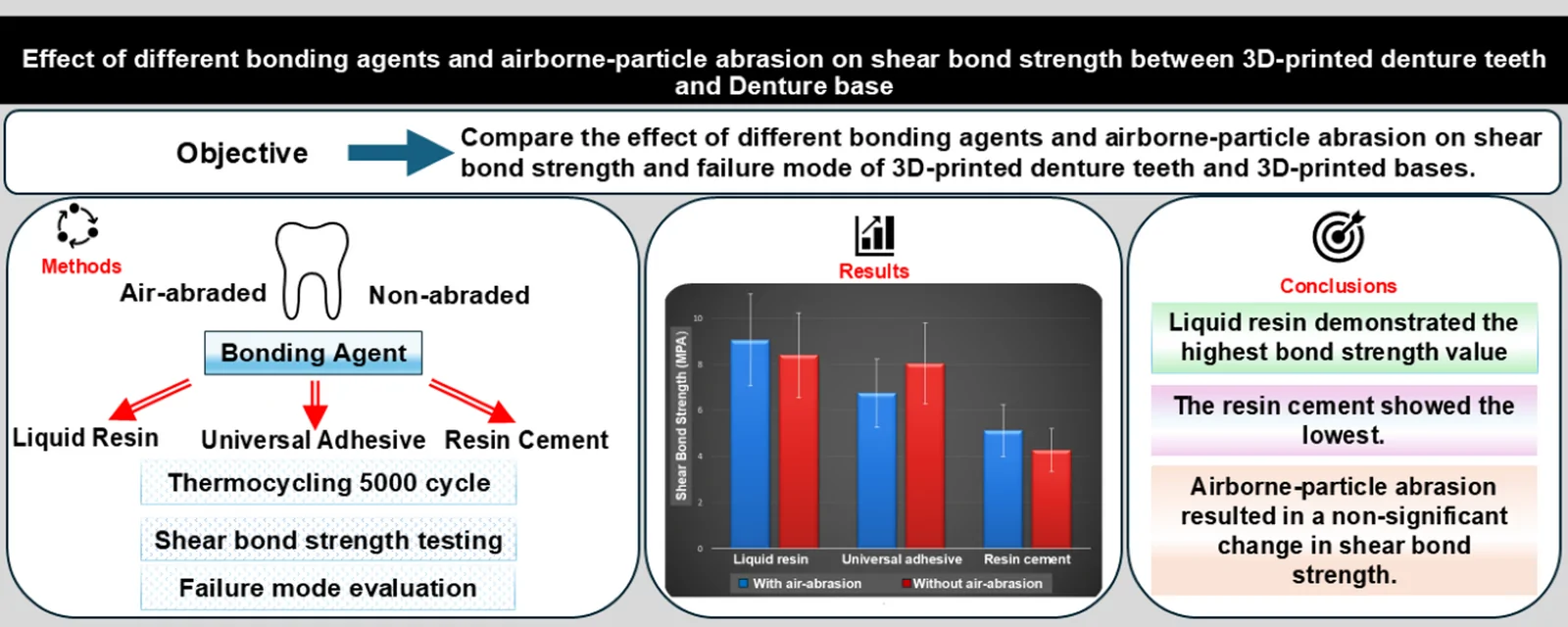

## Background

Since the 1950s, heat-polymerized polymethyl methacrylate (PMMA) resin has been the material of choice for complete denture fabrication; however, their workflow is a time-consuming, technique-sensitive laboratory procedure with many drawbacks [1]. To overcome these limitations, various materials and techniques have been introduced for complete denture construction [2–4]. More recently, computer-aided design and computer-aided manufacturing (CAD-CAM) have been used for complete denture fabrication, offering improved standardization, enhanced reproducibility, and reduced laboratory-related errors [5–7].

Digital dentures are fabricated either by milling from PMMA blocks or by three-dimensional (3D) printing, in which the denture base and the denture teeth are formed layer by layer from liquid, photo-polymerized resin [8, 9]. The use of prepolymerized PMMA blocks ensures a maximum degree of monomer conversion with favorable dimensional and mechanical properties [6]. However, the milling technique suffers from limitations in producing complex structures, which restrict the customization needed for certain dental applications. On the other hand, 3D printing offers greater design flexibility, with highly detailed patient-specific designs and lower cost, making it a promising alternative for complete denture fabrication [7, 10].

The bond strength between the denture teeth and denture base is a critical determinant of the structural integrity and functional durability of complete dentures. In conventionally fabricated dentures, debonding between the denture teeth and the denture base has been one of the complications encountered in complete dentures. It was found that it accounts for 22 to 30% of all denture repairs, with failures predominantly occurring in the anterior region [11]. Various surface treatments and bonding protocols, including mechanical abrasion, chemical conditioning using methyl methacrylate monomer, and other bonding agents, have been proposed to enhance adhesion between denture teeth and denture base [3, 12, 13]. Unlike conventional dentures, in which the teeth and base undergo simultaneous polymerization with chemical interfusion and copolymerization phenomena, digitally manufactured dentures are fabricated from prepolymerized or highly polymerized materials with minimally reactive surface [14–17]. So, the bonding mechanism between digitally fabricated denture teeth and base differs from the conventional ones [18]. Previous studies have evaluated different combinations of denture base and denture tooth materials, including milled or 3D-printed denture bases paired with prefabricated, milled, or 3D-printed teeth and different surface treatments [19–22]. Limited studies are available regarding bonding protocols between 3D-printed denture teeth and 3D-printed denture bases and the effect of different bonding agents and surface treatments on bond strength [23].

The three bonding protocols were selected to represent different clinically available approaches. The liquid resin for 3D printing of denture bases is chemically similar to the printed substrates and may promote bonding through surface softening and monomer diffusion [9]. The universal adhesive contains etching and coupling monomers that may contribute to micromechanical and chemical adhesion through the formation of a thin adhesive layer [24]. The self-adhesive resin cement uses acidic functional monomers that may promote chemical interaction with the substrates, together with mechanical interlocking without requiring a separate etching or priming step [25].

Therefore, this in vitro study aimed to evaluate the effect of the three bonding agents and airborne-particle abrasion on the shear bond strength between 3D-printed denture bases and tooth-colored substrates. The null hypotheses were as follows: (1) the type of bonding agent would not significantly affect shear bond strength between 3D-printed denture base and 3D-printed tooth-colored substrates; (2) airborne-particle abrasion would not significantly affect shear bond strength between 3D-printed denture base and 3D-printed tooth-colored substrates; and (3) no interaction would exist between the bonding agent and airborne-particle abrasion regarding the shear bond strength between 3D-printed denture base and 3D-printed tooth-colored substrates.

## Materials

### Study design and sample size

**Fig. 1:**
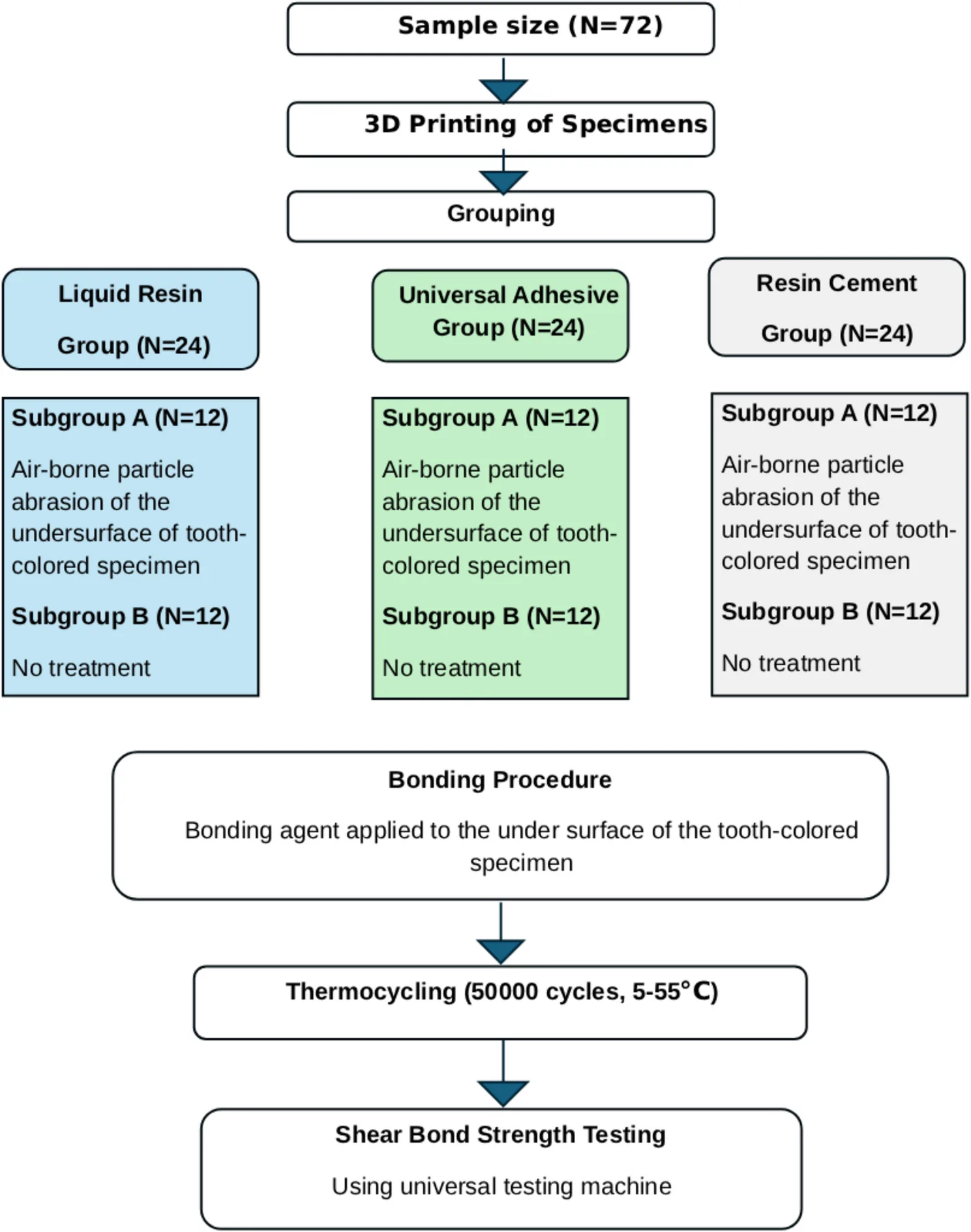
Study flowchart

The shear bond strength between 3D-printed denture bases and 3D-printed tooth-colored substrates with three different bonding agents representing different bonding protocols [26–31] (3D printing liquid base resin, universal adhesive, and resin cement) with and without airborne-particle abrasion surface treatment of the tooth-colored specimens was evaluated using a universal testing machine. The mode of failure was evaluated under a stereomicroscope (Fig. 1). Sample size was calculated for the comparison across the three bonding protocols (numerator degrees of freedom = 2) using an analysis software program (R version 4.3.2; R Foundation for Statistical Computing, Vienna, Austria; pwr package, pwr.anova.test function) for Windows by adopting an α = 0.05, β = 0.2 (power = 80%), and an anticipated large effect size (Cohen’s f = 0.4) based on the results of a previous study [32]. The calculation yielded a minimum of 22 specimens per group. To account for possible losses during thermocycling and mechanical testing, sample size increased to 24 specimens per group (*N* = 72). The calculation assumed independent, balanced groups with homogenous variance and was not designed to provide separate statistical power for the surface treatment main effect or the bonding protocol× surface treatment interaction. No specimens were lost during the study; all 72 specimens were fabricated, tested, and included in the final analysis.

### Specimen manufacturing

Specimens were designed using CAD software (Exocad DentalCAD; exocad GmbH, Darmstadt, Germany). The denture base specimen was designed as a block with dimensions of length 20 mm, width 20 mm, and 8 mm, and the denture tooth-colored specimen was designed as a cylinder with dimensions of 5 mm in diameter and 2.5 mm in height [33]. The designs were exported as STL files and fabricated using a 3D LCD printer (Phrozen Shuffle; Phrozen Tech Co., Ltd., Hsinchu, Taiwan) operating at a wavelength of 405 nm. The printer was calibrated prior to the specimens’ fabrication. Printing was performed at an irradiance of 7.0 mW/cm^2^, with an initial exposure time of 20 s and subsequent layers exposed for 2 seconds. The building platform temperature was maintained at 30 °C during the printing process. The printing parameters were set as follows: 90° printing orientation, no support structure (printed directly on the platform surface), and layer thickness of 50 μm was used for all the specimens. Denture specimens were printed from pink resin (NextDent Denture3D+, Classic Pink; NextDent B.V., Soesterberg, Netherlands), and the tooth-colored specimens were printed from tooth-colored resin (EPAX 3D Temporary Resin, shade 3; EPAX 3D, Morrisville, NC, USA). All materials were used within their commercial shelf life, and the relevant material information needed data is presented in Table 1.

**Table 1:** Comprehensive overview of and manufacturers’ reported compositions of materials used

Material	Pink resin	Tooth-colored resin	Universal adhesive	Self-adhesive resin
Brand name	NextDent Denture 3D+ (Classic Pink)	Epax 3D Temporary Resin (shade 3)	ONE COAT 7 UNIVERSAL	G-CEM Capsule
Manufacturer	3D Systems/NextDent B.V. (Soesterberg, Netherlands)	Epax 3D (Morrisville, NC, USA)	Coltene/Whaledent AG (Altstätten, Switzerland)	GC Corp. (Tokyo, Japan)
Chemical composition	Methacrylate-based photopolymer: ethoxylated bisphenol-A dimethacrylate (BIS-EMA), urethane dimethacrylate (UDMA), HEMA, SiO_2_ fillers, diphenyl(2, 4, 6-trimethylbenzoyl) phosphine oxide (photo-initiator), pigments	Methacrylate/acrylate-based photopolymer mixture: Epoxy acrylic oligomers, ethoxylated trimethylolpropane triacrylate, ethyl (2,4,6-trimethylbenzoyl) phenylphosphine photo-initiators, titanium oxide (TIO_2),_ shade matching inorganic pigments (EPAX 3D SDS Rev. 2021; components > 1% omitted by manufacturer)	Solvents (ethanol/water), methacrylate monomers, etching agents (10-MDP, polyacrylic acid), silica filler (components > 1% omitted by manufacturer)	4-MET (or 4-META per March 2016 SDS), phosphoric acid ester monomers, methacrylate monomers, fluoroaluminosilicate glass fillers, water, redox/photo-initiator system, stabilizers
Regulatory classification	Class II (21 CFR 872.3760)	Class II (21 CFR 872.3770/872.3690)	Class II (21 CFR 872.3200)	Class II (21 CFR 872.3275)
Manufacturer-stated intended use	Material for 3D printing of removable denture bases	Provisional dental restoration material (temporary crown, bridges and tooth-colored restorations)	Universal dental adhesives for direct composite/compomer restorations, indirect restoration bonding 9inlays, onlays, crowns, veneers), and cavity/tooth prep sealing	Dual-cure self-adhesive resin cement for the permanent luting of all-ceramic, resin, and metal-based inlays, onlays, crowns, bridges, and posts
Document source and revision (Safety Data Sheet (SDS) and/or Instruction For Use (IFU))	SDS: NextDent Denture 3D+, Rev. 2.0; issued January9, 2018; revised January 22, 2018; EU/REACH	SDS/Technical data sheet: EPAX 3D Temporary Resin, Rev. 2021	IFU: ONE COAT 7 UNIVERSAL, Rev. 03-2025	IFU: G-CEM Capsule, Rev. July 2025; SDS: Rev. March 2016
Processing/application method in the present study	LCD 3D printing at 50 μm layer thickness; followed by post printing isopropyl alcohol rinsing, air drying and UV post-polymerization	LCD 3D printing at 50 μm layer thickness; followed by post printing isopropyl alcohol rinsing, air drying and UV post-polymerization (405 nm wavelength)	Direct application (20 s. rub, 5 s. sir-dry, 10 s. photo-polymerization)	Capsule activation by auto-mixing, dual-polymerization (chemical and photo-polymerization)

Once printing was complete, the specimens were rinsed with 99% ethyl alcohol twice for 3 min each and air-dried for 15 min. Subsequently, they were placed in a post-polymerization unit (Phrozen Cure Luna; Phrozen Tech Co., Ltd., Hsinchu, Taiwan) equipped with 405 nm UV LEDs operating at an irradiance of 40 mW/cm^2^ at an ambient chamber temperature of25°C for 15 min to promote further polymerization of the printed specimens.

### Randomization and specimens’ allocation

Two batches of specimens (*N* = 72) were fabricated using 3D printing and physically pooled upon delivery from the fabrication laboratory. The diameter of each specimen was verified using a digital caliper to confirm that it met the planned dimensions. As the specimens could no longer be identified according to their batch of origin, the pooled specimens were considered batch-blind before randomization. Each specimen was given a unique number, and the numbers were placed in an opaque container and blindly drawn and assigned to three groups (*N* = 24), each further subdivided into two subgroups (A and B)—simple lottery method—each subgroup has 12 specimens. This procedure ensured that the original batch could not systematically affect group or subgroup allocation.

### Surface treatment

The bonding area was defined by positioning the 5-mm-diameter tooth-colored specimen on the denture base surface at the intended bonding area, and manual tracing of its circumference with a fine tip marker was done. The resulting circular area, corresponding to a nominal bonding area of 19.63 mm^2^, was isolated using Teflon tape to prevent the bonding agent from becoming in contact with the surrounding unbonded denture base surface.

Surface treatment was applied to the tooth-colored specimen, as its undersurface corresponds to the ridge-lap (intaglio) surface of a conventional denture tooth and is the surface intended for bonding to the denture base. In subgroup A, the undersurface of the tooth-colored specimen was air-abraded using 110 μm alumina for 10 s at 0.28 MPa air pressure, with the nozzle positioned approximately 10 mm from the surface at a 90° angle. Air abrasion for all the specimens was performed by the same trained operator to maintain consistency. Following abrasion, the specimens were cleaned using an ultrasonic cleaner in 99% isopropyl alcohol for 5 min and air-dried using oil-free air to ensure the removal of any remaining alumina particles. In subgroup B, the undersurface of the specimens remained untreated.

### Bonding protocols

The three experimental groups represented distinct bonding protocols. In the first group, denture base liquid resin (NextDent Denture3D+, Classic Pink; NextDent B.V., Soesterberg, Netherlands) was applied using a bonding brush to the undersurface of the tooth-colored specimen. The tooth-colored specimens were positioned perpendicular to the denture base specimen and manually stabilized under axial pressure by a single operator, and any excess material extruded beyond the confines of the bonding area was removed using a clean bond brush, followed by initial photopolymerization (Woodpecker LED-D; Guilin Woodpecker Medical Instrument Co., Ltd., Guilin, China) for 1 min; subsequently, the specimens were placed in a post-polymerization unit (Phrozen Cure Luna; Phrozen Tech Co., Ltd., Hsinchu, Taiwan) for 15 min to ensure complete polymerization of unreacted monomer.

In the second group, a universal adhesive (One Coat 7 Universal; Coltene/Whaledent AG, Altstätten, Switzerland) was applied with a microbrush to the undersurface of the tooth-colored specimen. The adhesive was rubbed on the surface for 20 s and then thinned with a gentle stream of oil-free air for 5 s in accordance with the manufacturer’s instructions. No additional bonding material was used. The tooth-colored specimen was subsequently positioned perpendicular to the denture base and manually stabilized as in the first group, and then the assembly was photopolymerized for 10 s (Woodpecker LED-D; Guilin Woodpecker Medical Instrument Co., Ltd., Guilin, China) (< 800Mw/cm^2^).

In the third group, a self-adhesive, dual-polymerizing methacrylate-based resin cement (G-CEM Capsule; GC Corp., Tokyo, Japan) was applied to the undersurface of the tooth-colored specimen. First the capsule was gently shaken before activation to homogenize its contents. The plunger was then fully depressed and maintained in this position for 2 s. The activated capsule was immediately transferred to an amalgamator (G8, Guilin Woodpecker Medical Instrument Co., Ltd., Guilin, China) and mixed for 10 s at approximately 4000 rotations per minute. Immediately after mixing, the cement was applied to the target area, and the tooth-colored specimen was then positioned perpendicular to the denture base and manually stabilized in the same manner as described for the other groups. The procedure was completed within the manufacturer-specified working time of 2 min and 30 s. The cement was initially photopolymerized for 2 s to reach the gel phase, allowing the removal of the excess cement. Then the circular bonding area was divided into 4 sections, with each section photo-polymerized individually for 10 s, followed by an additional 10-second exposure from the occlusal direction, resulting in a total final photopolymerization time of approximately 50 s. Following initial 2-second photopolymerization, the Teflon tape was carefully removed across all specimens. A unique code was assigned to each specimen in the 6 subgroups to facilitate blinding during shear bond strength testing and failure mode evaluation.

### Thermocycling

To mimic the repeated temperature fluctuations that may occur in the oral environment, the specimens were stored in distilled water at 37 °C and subjected to aging using a thermocycler (REUZEit Arctic A10B Refrigerated Circulator; model 1524101; REUZEit, Santa Fe Springs, CA, USA) for 5000 cycles with temperatures fluctuations between 5 and 55 °C, with each bath lasting for 30 s and 10-second transfer intervals. This regime was selected based on previous dental materials studies in which 5000 cycles were used as an accelerated aging regimen for evaluating the effects of thermocycling on material performance [35–37]. The samples were stored in distilled water for 24 h at 37 °C before shear bond strength testing.

### Evaluation of shear bond strength and mode of failure

Shear bond strength was evaluated using a universal testing machine (Instron 2710 − 115; Instron Corp., Norwood, MA, USA) equipped with a 5 KN load cell by a second operator experienced in mechanical testing to ensure standardization. Each specimen was mounted so that the chisel-like blade was aligned close to and parallel to the tooth-colored resin-denture base interface, and the crosshead speed was adjusted to 1 mm/min [8]. Force was applied until fracture occurred, and the maximum load at failure was recorded. Shear bond strength for each specimen was calculated using the following equation: Shear bond strength (MPa) = maximum load at failure (N) / nominal bonding area (19.63mm^2^).

**Fig. 2:**
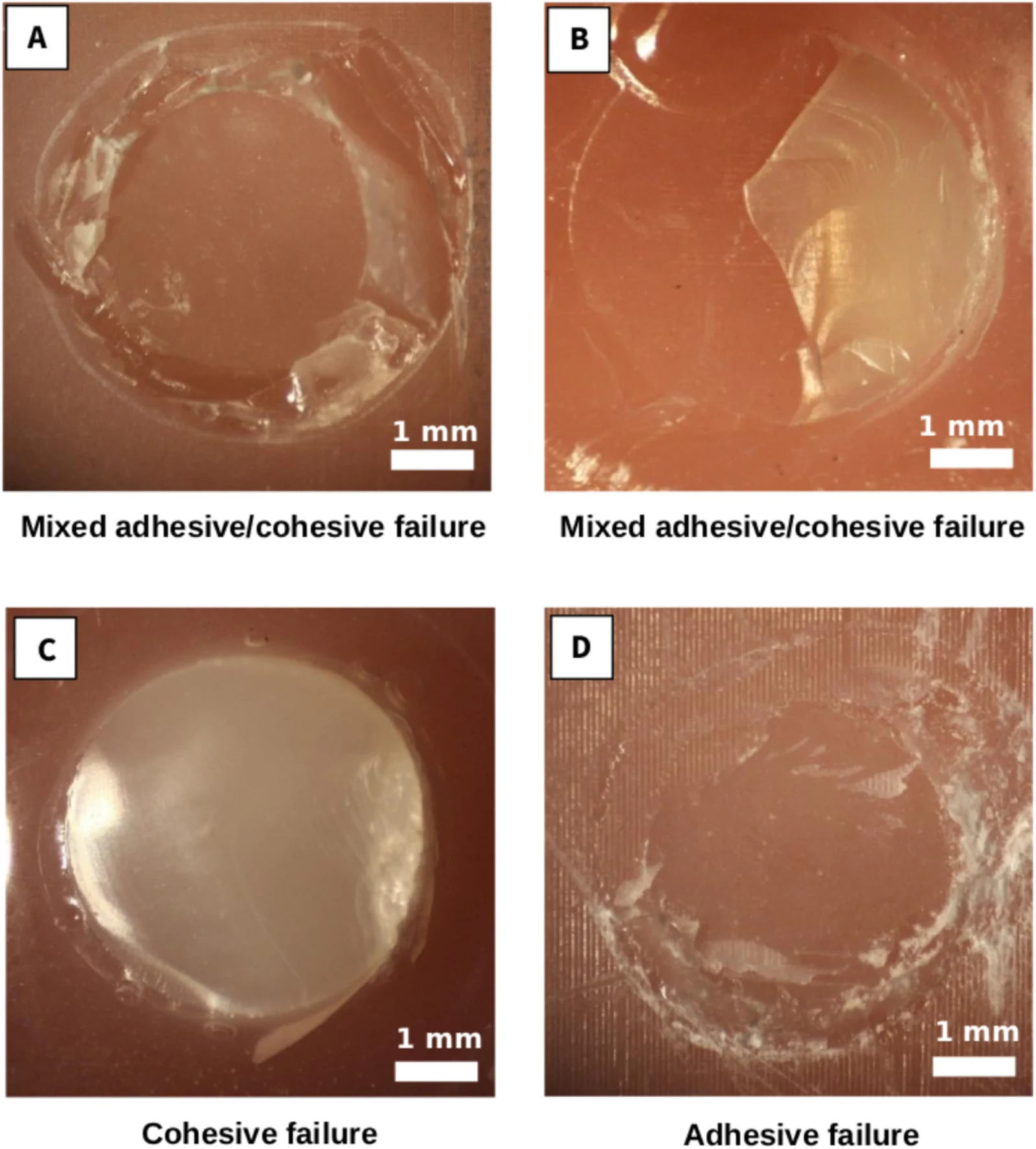
Representative stereomicroscopic images (×15 magnification; scale bar = 1 mm) illustrating the different forms of failure between tooth-colored resin and denture base resin after shear bond strength test. **A** and **B**: Mixed adhesive/cohesive failure, **C**: Cohesive failure and **D**: Adhesive failure

All the specimens were examined using the stereomicroscope (SZ40, Olympus, Tokyo, Japan) at 15x magnification to determine the mode of failure (Fig. 2). The mode of failure was classified according to the location and characteristics of the fracture observed under stereomicroscopic examination. An adhesive failure was defined as separation occurring at the tooth-colored resin-denture base interface without evidence of fracture within either substrate. A cohesive failure was identified when a fracture occurred within the tooth-colored or the denture base specimen, while the bonding interface remained intact. A mixed adhesive/cohesive failure was recorded when both interfacial separation and cohesive fracture were observed within either the denture base or tooth-colored specimen. The shear bond strength testing and failure mode assessment were performed independently by different examiners—one examiner for evaluating the shear bond strength and two for evaluating the mode of failure—each experienced in the respective procedure. All the examiners were blinded to specimen allocation and the corresponding bonding protocol.

### Statistical analysis

Data was analyzed using statistical analysis software (IBM SPSS Statistics, version 26; IBM Corp., Armonk, NY, USA). The Kolmogorov-Smirnov and Shapiro-Wilk tests were used to verify the normality of data distribution. Data showed parametric distribution; they were represented by mean and standard deviation (SD) values. Two-way analysis of variance (ANOVA) was used to assess the effect of the bonding agents and surface treatment and their interactions, and homogeneity of variance was verified using Levene’s test. Effect sizes for the Anova were expressed as partial eta-squared, with 95% confidence intervals calculated using noncentral F-distribution methods. Pairwise comparisons between the six groups were done utilizing t-tests with Bonferroni correction for multiple comparisons, with 95% confidence intervals for the mean differences calculated using the same Bonferroni adjustment applied to each corresponding family of comparisons. The distribution of failure modes among the liquid resin, universal adhesive, and resin cement groups was analyzed separately for specimens with and without air abrasion using the Fisher’s exact test, as appropriate based on the cell frequencies. The effect of air abrasion within each material group was similarly assessed using Fisher’s exact test. Inter-rater reliability of failure-mode assessment was evaluated using Cohen’s kappa (κ). The significance level was set at *P* ≤ 0.05.

## Results

The effect of different variables and their interactions on shear bond strength is presented in Table 2. Within the air-abraded groups (Table 3), a statistically significant difference was reported between all the groups (*P* < 0.001), with the highest mean value recorded in the liquid resin group, followed by the universal adhesive and the resin cement groups. Within the untreated groups, insignificant differences were reported between the liquid resin 8.39 ± 2.12 and universal adhesive 8.03 ± 2.09 groups, while the resin cement group exhibited a significantly lower mean value 4.27 ± 1.46 (*P* < 0.001). Intragroup comparison revealed no significant effect of airborne-particle abrasion on shear bond strength reported in all groups (*P* = 0.438, 95% CI (-1.01, 2.31); *P* = 0.130, 95% CI (-2.94, 0.39); and *P* = 0.315, 95% CI (0.82, 2.51) respectively).

**Table 2:** Effect of different variables and their interactions on shear bond strength (MPa)

Source	Type III Sum of Squares	df	Mean Square	F	Partial η^2^	95% CI for η^2^	Sig.
Type of bonding material	201.410	2	100.705	24.145	0.423	(0.268, 1.000)	< 0.001*
Surface Treatment	0.094	1	0.094	0.022	< 0.001	(0.000, 1.000)	0.881
Type of bonding material * Treatment	16.519	2	8.259	1.980	0.057	(0.000, 1.000)	0.146
Error	275.279	66	4.171				

Confidence intervals for Partial η^2^ are one-sided (upper bound fixed at 1.000)

*df *degree of freedom, *CI *Confidence intervals.

*; significant (*p* ≤ 0.05)

**Table 3:** Pairwise comparison of mean and standard deviation of shear bond strength (MPa) of 3D-printed denture base-tooth specimens across bonding agent groups and surface treatment subgroups

Treatment	Type of bonding material			*P*-value for bonding agent
	Liquid resin	Universal adhesive	Resin cement	
With air abrasion	9.04 ± 2.27Aa	6.75 ± 2.41Aab	5.12 ± 1.75Ab	< 0.001*
Without air abrasion	8.39 ± 2.12Aa	8.03 ± 2.09Aa	4.27 ± 1.46Ab	< 0.001*
*p*-value for surface treatment within each bonding protocol (CI for mean difference)	0.438 (-1.01, 2.31)	0.130 (-2.94, 0.39)	0.315 (-0.82, 2.51)	

Different lowercase letters within the same raw indicate significant difference at (*P* < 0.05) among bonding materials. Different uppercase letters within the same column indicate significant difference at (*P* < 0.05) between surface treatment conditions

*CI* Confidence intervals

*; significant (*P* ≤ 0.05)

Comprehensive pairwise comparisons among the six subgroups (Table 4), Bonferroni correction across all 15 pairwise comparisons, revealed that both air-abraded 9.04 + 2.27 and non-abraded 8.39 + 2.12 liquid resin subgroups and the non-abraded universal adhesive subgroup 8.03 + 2.09 demonstrated significantly higher bond strength than the air-abraded 5.12 + 1.75 and non-abraded 4.27 + 1.46 resin cement subgroups (*P* < 0.013 for all six comparisons; 95% confidence intervals for all pairwise differences are reported on Table 4). In contrast, none of the remaining pairwise comparisons demonstrated a statistically significant difference; this included comparisons between the air-abraded universal adhesive subgroup 6.75 + 2.41 and either liquid resin subgroups (*P* = 0.117 and *P* = 0.806), as well as comparisons between the air-abraded universal adhesive subgroup and either resin cement subgroups (*P* = 0.812 and *P* = 0.062) and between the liquid resin subgroups (*P* = 1.000).

**Table 4:** Comprehensive pairwise comparison of mean shear bond strength (MPa) values across all six experimental groups

Group 1	Group 2	Mean 1 (MPa)	Mean 2 (MPa)	95% CI for differences	*P*-value
Liquid resin (with air abrasion)	Liquid resin (without air abrasion)	9.04 ± 2.27a	8.39 ± 2.12a	(-1.89, 3.19)	1.000
Liquid resin (with air abrasion)	Universal adhesive (with air abrasion)	9.04 ± 2.27a	6.75 ± 2.41a	(-0.25, 4.83)	0.117
Liquid resin (with air abrasion)	Universal adhesive (without air abrasion)	9.04 ± 2.27a	8.03 ± 2.09a	(-1.53, 3.55)	1.000
Liquid resin (with air abrasion)	Resin cement (with air abrasion)	9.04 ± 2.27a	5.12 ± 1.75b	(1.38, 6.46)	< 0.001
Liquid resin (with air abrasion)	Resin cement (without air abrasion)	9.04 ± 2.27a	4.27 ± 1.46b	(2.23, 7.31)	< 0.001
Liquid resin (without air abrasion)	Universal adhesive (with air abrasion)	8.39 ± 2.12a	6.75 ± 2.41a	(-0.90, 4.18)	0.806
Liquid resin (without air abrasion)	Universal adhesive (without air abrasion)	8.39 ± 2.12a	8.03 ± 2.09a	(-2.18, 2.90)	1.000
Liquid resin (without air abrasion)	Resin cement (with air abrasion)	8.39 ± 2.12a	5.12 ± 1.75b	(0.73, 5.81)	0.003
Liquid resin (without air abrasion)	Resin cement (without air abrasion)	8.39 ± 2.12a	4.27 ± 1.46b	(1.58, 6.66)	< 0.001
Universal adhesive (with air abrasion)	Universal adhesive (without air abrasion)	6.75 ± 2.41a	8.03 ± 2.09a	(-3.82, 1.26)	1.000
Universal adhesive (with air abrasion)	Resin cement (with air abrasion)	6.75 ± 2.41a	5.12 ± 1.75a	(-0.90, 4.17)	0.812
Universal adhesive (with air abrasion)	Resin cement (without air abrasion)	6.75 ± 2.41a	4.27 ± 1.46a	(-0.06, 5.02)	0.062
Universal adhesive (without air abrasion)	Resin cement (with air abrasion)	8.03 ± 2.09a	5.12 ± 1.75b	(0.37, 5.45)	0.013
Universal adhesive (without air abrasion)	Resin cement (without air abrasion)	8.03 ± 2.09a	4.27 ± 1.46b	(1.22, 6.30)	< 0.001
Resin cement (with air abrasion)	Resin cement (without air abrasion)	5.12 ± 1.75a	4.27 ± 1.46a	(-1.70, 3.38)	1.000

Different small letters indicate significant difference at (*P* < 0.05) among means in the same row. Bonferroni correction was applied across all pairwise comparisons

The distribution of failure modes among the experimental groups presented in Table 5. Intergroup comparison showed that there was a statistically significant difference in the distribution of failure modes among the liquid resin, universal adhesive, and resin cement groups, both with air abrasion and without air abrasion (*P* < 0.001 for both comparisons). With air abrasion, adhesive failures predominated in the resin cement group (100.0%), whereas cohesive failures were most frequent in the liquid resin group (91.7%). In the universal adhesive group, adhesive, cohesive, and mixed failures accounted for 50.0%, 16.7%, and 33.3%, respectively. Without air abrasion, adhesive failure occurred in 100.0% of the universal adhesive and resin cement groups, while the liquid resin group showed 25% cohesive and 75% mixed failures (*P* < 0.001). For the intragroup comparison, the distribution of failure modes also differed significantly between specimens with and without air abrasion in the liquid resin group (*P* < 0.001) and the universal adhesive group (*P* = 0.010). The resin cement group showed no variation in failure mode, with all failures classified as adhesive regardless of air abrasion.

**Table 5:** Distribution of failure modes among the groups with and without air abrasion

		Group						*P* value
		Liquid resin		Universal adhesive		Resin cement		
		Count	Column *N* %	Count	Column *N* %	Count	Column *N* %	
With air abrasion	Adhesive	1	8.3%	6	50.0%	12	100.0%	< 0.001*
	Cohesive	11	91.7%	2	16.7%	0	0.0%	
	Mixed adhesive/cohesive	0	0.0%	4	33.3%	0	0.0%	
Without air abrasion	Adhesive	0	0.0%	12	100.0%	12	100.0%	< 0.001*
	Cohesive	3	25%	0	0.0%	0	0.0%	
	Mixed adhesive/cohesive	9	75%	0	0.0%	0	0.0%	
*P* value		< 0.001*		0.01*		****		

Mixed adhesive/cohesive failure refers to specimens exhibiting both adhesive separation and cohesive fracture within either the denture tooth or the denture base

*; significant (*P* ≤ 0.05)

****; not computable, failure mode is both groups is identical

In Table 6, Cohen’s kappa demonstrated statistically significant agreement for the assessment of failure modes across all material and air-abrasion conditions (*P* < 0.05). The kappa values ranged from 0.636 to 1.000, indicating substantial to almost perfect agreement. The highest agreement was observed for the universal adhesive without air abrasion and both resin cement conditions (κ = 1.000, *P* < 0.001). The liquid resin with air abrasion showed substantial agreement (κ = 0.636, *P* < 0.001), while the liquid resin without air abrasion showed substantial agreement (κ = 0.800, *P* = 0.018). The universal adhesive with air abrasion demonstrated almost perfect agreement (κ = 0.860, *P* < 0.001).

**Table 6:** Inter-rater reliability of failure mode assessment using Cohen’s Kappa in the different groups

Kappa	Value	Exact Significance
Liquid resin with air abrasion	0.636	< 0.001*
Liquid resin without air abrasion	0.800	0.018*
Universal adhesive with air abrasion	0.860	< 0.001*
Universal adhesive without air abrasion	1.00	< 0.001*
Resin cement with air abrasion	1.00	< 0.001*
Resin cement with air abrasion	1.00	< 0.001*

*; significant agreement as *P* < 0.05

## Discussion

The first null hypothesis was rejected, as the effect of the bonding protocol on shear bond strength was statistically significant, while the second null hypothesis was accepted, as the effect of airborne-particle abrasion was statistically insignificant. The third null hypothesis was accepted, as no significant interaction was identified between bonding protocol and surface treatment. The advance of 3D printing technology has revolutionized and made far-reaching changes in various fields, including dentistry. One of the significant applications of this technology is in complete denture fabrication. The integration of 3D-printed teeth with 3D-printed denture bases offers numerous advantages, including precision, customization, and efficiency. However, the durability and functionality of these dentures depend on other factors, including the shear bond strength between the 3D-printed teeth and the denture base. Despite the introduction of 3D manufacturing technology for denture fabrication, few investigations are available to verify the bonding protocols and their effect on bond strength between 3D-printed denture teeth and the denture base [8, 14, 15, 32].

Assessing the shear bond strength involves subjecting the bond interface to a perpendicular force until failure occurs. This mechanical test provides a valuable insight into the bond’s integrity and enables comparison of the bonding protocol under standardized laboratory conditions. Understanding these factors may contribute to the development of more reliable bonding strategies for 3D-printed substrates [16, 34]. The present study demonstrated no significant overall effect of airborne-particle abrasion on shear bond strength, consistent with the absence of a significant effect within any individual group. The bonding agent-surface treatment interaction was also not statistically significant. Several studies have reported that airborne-particle abrasion prior to the application of the bonding agent improves shear bond strength [4, 11, 13, 14]. However, these investigations assess either conventionally processed materials or combinations involving conventional substrates, which have lower density and allow effective micromechanical retention. In contrast, the present study assessed 3D-printed substrates and three bonding agents with different material properties and application characteristics; these, together with the distinct structural characteristics of these substrates, contribute to the differences observed among the investigated protocols.

Liquid resin was chosen for its chemical similarity with 3D-printed substrates and its ability to undergo copolymerization. It is composed mainly of unreacted methacrylate monomers that can directly interact with the surface of 3D-printed substrates. Previous studies have proposed that the application of resin monomers to polymeric substrates may induce surface softening or swelling—acting as a solvent—allowing mutual diffusion of the resin components by what is known as the chemical dissolution and swelling mechanism. Subsequent polymerization may facilitate interfacial integration between the resin layers, resulting in the formation of a continuous methacrylate network; however, the extent of such integration was not directly evaluated in this study [9]. This proposed interfacial interaction may contribute to the superior bond strength observed in this group compared with the other two groups [4, 12, 32]. Airborne-particle abrasion in this group did not result in a significant increase in bond strength; however, a slight improvement was observed, which was also associated with a qualitative shift in the mode of failure, as the non-abraded subgroup showed predominantly mixed adhesive/cohesive failure (75%), whereas cohesive failures became more frequent following air abrasion (91.7%), with mixed failures no longer observed. This slight numerical increase in bond strength and the redistribution in mode of failure may be associated with the changes in the surface roughness and surface energy following airborne-particle abrasion, which have been reported to affect wettability and facilitate monomer infiltration [20]. However, in adhesive systems where bonding may involve greater contribution from chemical interactions and polymerization, as with liquid resin, the effect of micromechanical retention may be limited, which may explain the minimal impact of surface treatment on the bond strength. Similar findings have been reported for highly cross-linked materials [15, 18–20]. Han et al. [9] observed that airborne-particle abrasion of milled denture teeth resulted in a smoother surface, as the dense polymer network resisted the formation of effective undercuts. Likewise, Alhomsi et al. [20] and Radhi Neamah et al. [21] reported that sandblasting did not improve the bond and attributed this to the high density of milled resin material. In a similar context, post-polymerized 3D-printed resin has a dense, highly cross-linked polymer network, which may limit the effect of mechanical surface treatment [22,38].

Manufacturer’s documentation reports that the G-CEM capsule is a dual-polymerizing material containing functional acidic monomers—specifically 4-methacryloyloxyethyl trimellitic acid (4-MET; also reported as 4-META in the older safety data sheet documentation) and phosphoric ester monomers—alongside methacrylate monomers, water, and fluoroaluminosilicate glass fiber. Also, for the ONE COAT 7 universal adhesive, it was reported that it contains functional monomers and acidic etching agents, including 10-methacryloyloxydecyl dihydrogen phosphate (10-MDP)-containing etching agents and polyacrylic acid, alongside methacrylate monomers, silica fillers, and a water/ethanol solvent system. The presence of these functional monomers has been reported to participate in chemical interactions with the polar groups on suitable substrate surfaces [25]. As the present study compared complete commercial bonding protocols and did not isolate individual monomers or characterize interfacial chemistry, the lower bond strength observed with this protocol cannot be attributed to a specific functional monomer and may instead reflect the combined characteristics of the bonding protocol, including resin matrix, filler loading, solvent content, viscosity, curing mode, and interfacial layer thickness.

The universal adhesive protocol exhibited 100% of adhesive failure mode in the non-abraded subgroup, while following airborne-particle abrasion, adhesive failures accounted for 50% of the specimens, while cohesive and mixed adhesive/cohesive failures accounted for 16.7% and 33.3% respectively. Thus, although airborne-particle abrasion was associated with a numerical reduction in bond strength in this protocol, it also produced a different distribution in mode of failure, including failure in cohesive components.

The slight increase in bond strength observed with G-CEM after airborne-particle abrasion may be related to the creation of micro-mechanical irregularities and exposure of additional polar sites, which may enhance the interaction with the resin cement [29]. However, the dense, highly cross-linked polymer structure may limit the effectiveness of airborne-particle abrasion, particularly when combined with the relatively high viscosity of the cement, which may restrict its infiltration into micro-irregularities, reducing the overall effectiveness of surface treatment [30]. The exclusive failure pattern observed in the resin cement subgroups was consistent with the lower shear bond strength observed for this bonding protocol.

One finding worth noting is that the mean shear bond strength of the non-abraded universal adhesive subgroup showed statistically comparable results to those of the liquid resin subgroups, but this statistical similarity does not, by itself, support a clinical recommendation or durability inference, as, the failure mode analysis revealed a clear difference between the two bonding protocols. While the liquid resin subgroups exhibited predominantly failure modes with a cohesive component, all the non-abraded universal adhesive subgroup specimens exhibited adhesive failure. This difference failure mode distribution, despite the statistically comparable bond strength may indicate weaker interfacial strength. In addition, this study did not assess mechanical fatigue or occlusal loading.

In contrast to the other groups, the combination of airborne-particle abrasion and universal adhesive resulted in a numerical reduction in shear bond strength. Although this reduction is not statistically significant, it may reflect a different response in the adhesive protocol to surface treatment. This is consistent with the findings of Arslan et al. [23], who reported similar outcomes when they evaluated the effect of tribochemical sandblasting combined with universal bonding adhesive on the bond strength of 3D-printed substrates. The authors attributed these results to disruption in the resin matrix caused by airborne-particle abrasion, which reduces the bonding affinity of the surface, interfering with the adhesive’s ability to form effective chemical interactions. Furthermore, they concluded that adhesive systems where the bond mechanism relies primarily on the substrate’s surface chemistry achieve favorable results with untreated substrates. Despite the proposed explanations for the effect of airborne-particle abrasion within each group, the underlying cause for its inconsistent behavior across the different groups remains unclear.

The three bonding protocols evaluated involved different curing regimens rather than a single standardized curing time, reflecting the distinct characteristics and intended applications of the materials used. Light transmission and polymerization can vary with material thickness and composition; therefore, the curing requirements of a bulk resin or a resin cement may differ from those of a thin adhesive layer [23]. Similarly, a uniform bonding layer was not imposed because this would have required modification of the individual application techniques. The resulting interfacial layer was consequently considered an inherent characteristic of each bonding protocol rather than an independent experimental variable. Accordingly, each material was polymerized using the curing procedure assigned to its respective protocol, and the study compared the overall shear bond strength of the three bonding protocols under consistent experimental conditions rather than the effect of a standardized bonding layer thickness or curing regimen. The LED curing units used (Woodpecker LED-D and Phrozen Cure Luna) provide light output within the visible-light range relevant to the activation of the photoinitiator used in methacrylate-based dental resins, adhesives, and cements.

Thermocycling was used to simulate the repeated temperature changes that occur in the oral environment. Although this approach is supported in literature as a valuable method for reproducing clinical temperature fluctuation and simulating aging, its influence on shear bond strength between denture teeth and denture bases remains inconclusive, as previous studies have reported inconsistent findings [14, 35–37].

### Limitations and future scope

This study was limited by its in vitro design using a single surface treatment applied only to the tooth-colored specimen-bonding surface and the assessment of bond strength without occlusal loading or the use of a positioning jig and standardized clamping force during bonding. Another limitation is that the tooth-colored resin used in this study was not documented as a commercially validated denture-tooth material for definitive intraoral use. Furthermore, this study did not include a non-thermocycled control group; therefore, the impact of thermocycling on shear bond strength could not be determined, raising questions about the potential influence of aging on bonding failures in 3D-printed dentures. The thermocycling protocol did not incorporate mechanical fatigue loading or pH variation, all of which may influence hydrolytic degradation and long-term bond stability under clinical conditions. Further investigations should evaluate commercially validated denture-tooth resins, various bonding protocols, and surface treatments using a standardized jig with controlled bonding loads, under mechanical loading and thermal aging conditions to optimize bonding strategies for 3D-printed dentures to enhance their durability and strengthen the clinical applicability of the findings. Regarding the observed dissociation between failure mode and mean shear bond strength for liquid resin and universal adhesive protocols, whereby airborne-particle abrasion significantly altered failure patterns without significantly affecting shear bond strength, needs further investigations each bonding protocol at the interfacial level including fractographic and surface characterization methods, together with large sample sizes, to help determine whether this findings reflect a subtle influence of airborne-particle abrasion on the failure mode or the limited sensitivity of shear bond strength testing to detect such differences.

## Conclusions

Within the limitations of this in vitro study and under the specific material and processing conditions evaluated, the shear bond strength between the 3D-printed denture base and tooth-colored specimens differed significantly according to the bonding protocol. Denture base liquid resin demonstrated the highest bond strength values, irrespective of surface treatment, while the self-adhesive resin cement showed the lowest. Although airborne-particle abrasion of the tooth-colored specimen-bonding surface resulted in no statistically significant change in shear bond strength within any of the evaluated bonding protocols, failure mode analysis revealed a significant alteration in the distribution of the failure modes for the liquid resin and universal adhesive protocols. 

## Data Availability

Availability of data and materials: Available upon request from the corresponding author.

## Abbreviations

3D: Three-dimensional
PMMA: Polymethyl methacrylate
CAD-CAM: Computer-aided design and computer-aided manufacturing
